# Research Advances of Extrachromosomal Circular DNA in Breast Cancer

**DOI:** 10.1002/cam4.71285

**Published:** 2025-10-06

**Authors:** Xiaodan Zhu, Yiyang Qian, Quan Tang, Jiahui Li, Yuqing Geng, Shixia Gong, Chunhui Jin

**Affiliations:** ^1^ Wuxi Hospital Affiliated to Nanjing University of Chinese Medicine Wuxi China

**Keywords:** biological function, biomarker, breast cancer, extrachromosomal circular DNA (ecDNA), targeted therapy, therapeutic strategies

## Abstract

**Background:**

This article presents an extensive review of the advancements in research concerning extrachromosomal circular DNA (ecDNA) within the context of breast cancer. As a distinct form of DNA, ecDNA is critically involved in the initiation, progression, diagnosis, and treatment of breast cancer.

**Methods:**

The article provides a comprehensive analysis of the mechanisms underlying the formation of ecDNA, highlighting factors such as aberrant DNA damage repair and chromosomal rearrangements. It further examines the biological roles of ecDNA in augmenting oncogene expression, fostering tumor heterogeneity, and facilitating immune evasion.

**Results:**

Epidemiological studies indicate significant variability in the distribution of ecDNA across different breast cancer subtypes, with a notable association with invasive subtypes, such as triple‐negative breast cancer. The presence of ecDNA is linked to poor prognosis and treatment resistance in patients. Current detection technologies for ecDNA include molecular biology and imaging techniques, and its detectability in plasma underscores its potential as a biomarker for liquid biopsy.

**Conclusions:**

The development of ecDNA‐targeted therapies and their integration with immunotherapy strategies offers promising new avenues for breast cancer treatment. Despite challenges such as incomplete elucidation of mechanisms, standardization of detection methods, and ethical considerations, ecDNA remains a valuable biomarker and therapeutic target in breast cancer. Future research is anticipated to advance its clinical transformation and application in individualized treatment.

**Abbreviations**
AI: artificial intelligence
AUC: area under the curve
CCND1: cyclin D1
Circle‐seq: circular DNA sequencing
CRISPR: clustered regularly interspaced short palindromic repeats
dCas9: catalytically “dead” Cas9
DDR: DNA damage response
DMs: double minute chromosomes
DSB: double‐strand break
eccDNA: extrachromosomal circular DNA
eccMIR: extrachromosomal circular DNA carrying miRNA
ecDNA: extrachromosomal DNA
EGFR: epidermal growth factor receptor
ER: estrogen receptor
ERBB2: erythroblastic leukemia viral oncogene homolog 2 (HER2)
FISH: fluorescence in situ hybridization
gRNA: guide RNA
HSGs: hotspot genes
HU: hydroxyurea
LADs: lamina‐associated domains
ML: Machine Learning
MMEJ: microhomology‐mediated end joining
MYC: MYC proto‐oncogene
NHEJ: nonhomologous end joining
ORC: origin recognition complex
POLθ: DNA polymerase theta
PR: progesterone receptor
qPCR: quantitative polymerase chain reaction
RCA: rolling circle amplification
SPRi: surface plasmon resonance imaging
TNBC: triple‐negative breast cancer

## 1 Overview of Extrachromosomal Circular DNA

Extrachromosomal DNA (ecDNA) specifically refers to large extrachromosomal particles existing in cancer cells and belongs to the functional subclass of extrachromosomal circular DNA (eccDNA) [1]. It can be detected in approximately 17% of cancers [2]. ecDNA is relatively large in size, reaching several million base pairs (at the Mb level) [3], and it maintains a relatively stable architecture characterized by unique topological and genetic features [4]. Research has demonstrated that ecDNA harbors full or partial gene sequences [2, 5] and can self‐replicate. This capacity enables ecDNA to play a pivotal role in the regulation of gene expression and the evolution of tumor cells. For instance, in various cancer cell lines, due to the lack of centromeres and telomeres, ecDNA leads to copy number heterogeneity in progeny cells through nonrandom allocation during cell division, thereby driving the evolution and adaptation of tumors [6, 7].

## 2 Formation Mechanisms and Biological Functions of ecDNA in Breast Cancer

In breast cancer, the mechanisms underlying ecDNA formation are intricate and involve a multitude of cellular processes. Aberrant repair of DNA double‐strand breaks is a significant contributor to ecDNA formation. Specifically, when DNA sustains double‐strand breaks, error‐prone repair pathways such as nonhomologous end joining (NHEJ) and microhomology‐mediated end joining (MMEJ) can lead to the circularization of chromosomal fragments, thereby generating ecDNA [8]. The high transcriptional activity caused by the open chromatin within ecDNA triggers persistent DNA double‐strand breaks (DSBS), which rely on the alt‐NHEJ pathway (LIG3/POLθ‐mediated) for repair to maintain cyclization, forming a vicious cycle of “mutation‐evolution” [9]. Additionally, chromosomal rearrangements and gene amplification are closely linked to ecDNA formation. Studies have demonstrated that in breast cancer cells, chromosomal regions harboring critical genes, such as Erythroblastic Leukemia Viral Oncogene Homolog 2 (ERBB2) and Cyclin D1 (CCND1), may undergo rearrangements, with the resultant fragments detaching from chromosomes to form ecDNA [10]. Genomic instability and chromatin remodeling are major contributors to ecDNA formation. Aberrant epigenetic changes, such as altered DNA methylation, further exacerbate this process. For example, in the ER^−^/PR^−^/HER2^+^ subtype of breast cancer, global DNA hypomethylation provides conditions for the formation of ecDNA by affecting chromatin stability and increasing the risk of DNA breaks and abnormal rearrangements. Meanwhile, the hypomethylation regions are mostly located in the late replication regions of the genome or the nuclear lamellar association domains (LADs), which themselves are prone to replication errors and breaks, further accelerating the generation of ecDNA [11].

**FIGURE 1:**
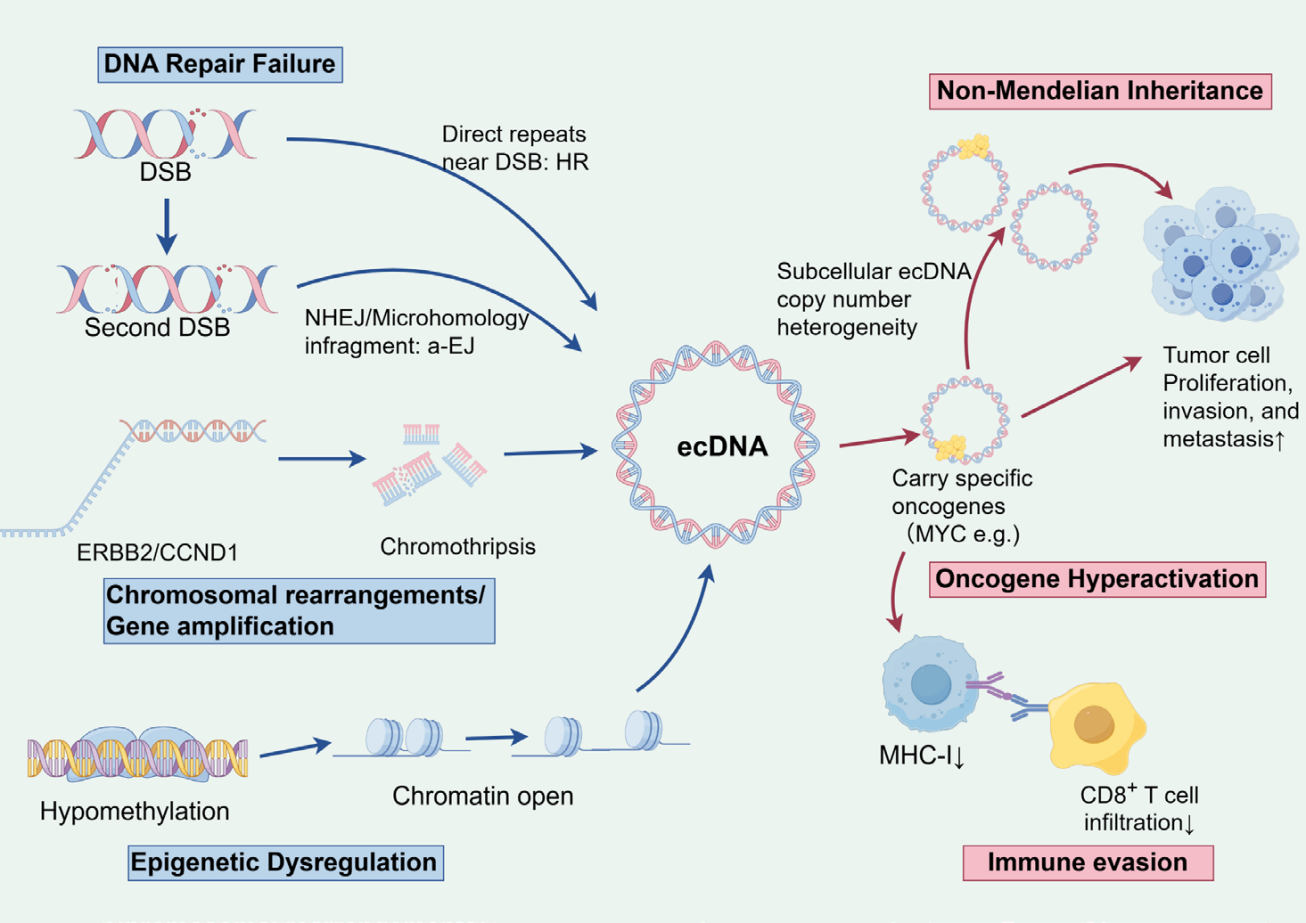
Integrated mechanisms and oncogenic functions of ecDNA in breast cancer. In breast cancer, the failure of DNA DSB repair pathways, such as NHEJ or microhomology‐mediated a‐EJ, leads to the circularization of chromosomal fragments, forming ecDNA. Large‐scale genomic rearrangements like chromothripsis, often involving oncogenes such as ERBB2 or CCND1, and epigenetic dysregulation, notably global DNA hypomethylation promoting open chromatin, are key mechanisms driving ecDNA biogenesis. Once formed, ecDNA exerts potent oncogenic functions: It carries specific oncogenes like MYC, enabling their massive hyperactivation due to its open chromatin architecture and super‐enhancers. ecDNA undergoes non‐Mendelian inheritance during cell division, resulting in significant subcellular copy number heterogeneity among daughter cells, which accelerates tumor evolution and the expansion of therapy‐resistant clones. Furthermore, ecDNA contributes to immune evasion by downregulating MHC‐I molecules, resulting in reduced CD8^+^ T cell infiltration into the tumor, thereby facilitating escape from immune surveillance. a‐EJ, alternative end‐joining; CCND1, Cyclin D1; CD8, Cluster of Differentiation 8; DSB, double‐strand break; ecDNA, extrachromosomal DNA; ERBB2, Erb‐B2 Receptor Tyrosine Kinase 2; MHC‐I, major histocompatibility complex class I; MYC, MYC Proto‐Oncogene; NHEJ, nonhomologous end joining.

ecDNA plays several pivotal biological roles in breast cancer. Firstly, ecDNA can facilitate tumor development by enhancing gene expression. Oncogenes located on ecDNA, such as MYC, due to their unique structure and open chromatin state, contribute to the accumulation of transcription factors and their interaction with proteins, including transcription factors. Additionally, the super enhancers present on ecDNA can activate oncogenes across different molecules [12]. This results in oncogene expression levels that are 30–50 times higher than those observed with chromosomal integration, thereby achieving high‐level transcription, increasing oncoprotein expression, and promoting tumor cell proliferation, invasion, and metastasis [13, 14, 15]. Recent studies have demonstrated that ecDNA can initiate multiple rounds of replication during the early S phase by hijacking the host ORC complex, thereby circumventing the temporal regulatory constraints of chromosomal DNA and achieving efficient amplification [7, 9]. Secondly, ecDNA significantly exacerbates the heterogeneity of tumor cells. The characteristic of nonrandom allocation results in substantial variations in ecDNA copy numbers among progeny cells, with differences exceeding tenfold, thereby facilitating the proliferation of drug‐resistant clones and enabling tumors to swiftly adapt to external stressors [3, 9, 16]. Notably, tumor environments often exhibit the coexistence of high and low ecDNA clone groups. Among these, low ecDNA clones function as an “evolutionary reserve” capable of rapid expansion under therapeutic pressure, thereby contributing to drug resistance [17]. The stability of GBM39‐EC cells maintained under glucose restriction through low ecDNA subsets, along with the development of drug resistance via reversible loss of EGFR‐ecDNA during anti‐EGFR treatment, provides compelling evidence of their dynamic evolutionary capacity [18]. Thirdly, ecDNA may facilitate immune evasion. The presence of ecDNA is closely related to the genomic instability of tumor cells. This instability may inhibit the recognition and attack capabilities of the immune system by altering the tumor microenvironment [2, 15, 19]. ecDNA can frequently amplify immunomodulatory and inflammation‐related genes, regulating lymphocyte‐mediated immunity and immune effector processes. Meanwhile, research data show that ecDNA carrying immunomodulatory genes is associated with reduced tumor T cell infiltration [2]. Research indicates that ecDNA harboring specific genes can modify tumor cell surface antigen expression, diminishing immune system recognition and attack, thus enabling tumor cells to evade immune surveillance [20]. In addition, ecDNA can also regulate the activity of immune cells through interaction with immune checkpoint molecules, thereby promoting the immune escape of tumors [21, 22] (Figure 1).

## 3 Epidemiological Studies of ecDNA in Breast Cancer

The epidemiological characteristics of ecDNA in breast cancer patients have attracted increasing scholarly interest. Research indicates that ecDNA is relatively prevalent in breast cancer tissues. Circle‐seq analysis conducted on 19 breast cancer tissues and 17 adjacent normal tissues revealed that ecDNA is distributed across all chromosomes and is enriched at seven hotspot genes associated with breast cancer [23]. The abundance of ecDNA varies significantly among different breast cancer cell lines (with a positive rate as high as 40%). For example, the triple‐negative MDA‐MB‐231 cell line exhibits significantly higher levels of ecDNA compared to the Luminal‐A MCF‐7 cell line, which may correlate with differences in invasiveness and metastatic potential [24]. Additionally, ecDNA can be detected in the plasma of breast cancer patients, with features partially overlapping those of tumor‐derived ecDNA, suggesting its potential as a noninvasive biomarker for diagnosis and monitoring [25].

## 4 Distribution of ecDNA Across Breast Cancer Subtypes

Breast cancer subtypes are characterized by distinct biological behaviors and prognostic outcomes, with ecDNA distribution patterns reflecting these differences. Studies indicate that aggressive triple‐negative breast cancers (TNBCs), known for their poor prognoses, tend to possess elevated levels of ecDNA and may contain subtype‐specific ecDNA variants [24]. An analysis of primary tumors and corresponding lymph node metastases from 19 breast cancer patients revealed that, although the ecDNA in metastatic sites shared chromosomal origins with primary tumors, there was selective enrichment of certain miRNA‐associated ecDNAs (such as miR‐6730 and miR‐548AA1) in the metastases, suggesting a role for ecDNA in metastatic progression [26]. Furthermore, ecDNA profiles associated with genomic repetitive sequences vary between hormone receptor‐positive and ‐negative subtypes, potentially contributing to their distinct pathogenesis and clinical behaviors [25].

## 5 Correlation Between ecDNA and Breast Cancer Prognosis

ecDNA is closely related to the prognosis of breast cancer, and its presence is significantly associated with enhanced tumor invasiveness, treatment resistance, and shortened patient survival period [2]. Studies have shown that breast cancer patients carrying specific ecDNA (such as MYC, EGFR, and other oncogenes) often have a poorer prognosis, and their 5‐year overall survival rate may be reduced by up to 35% [3]. For instance, in some breast cancer patients, the amplification of oncogenes on ecDNA is associated with a higher tumor stage, a 40% to 60% increased risk of metastasis, and a shortened overall survival period of the patients [3]. Studies on breast cancer cell lines have found that highly invasive cell lines, such as triple‐negative breast cancer subtypes, are rich in ecDNA, suggesting that ecDNA may promote tumor progression and thereby affect patient prognosis [24]. Furthermore, in patients who have received targeted therapy and cytotoxic chemotherapy, the detection of ecDNA is associated with tumor recurrence and metastasis, indicating that ecDNA may affect the long‐term survival of patients by mediating treatment resistance (increasing the drug resistance rate by 40% to 60%), suggesting that ecDNA may be involved in the treatment resistance process of breast cancer and influence the long‐term survival of patients [2].

## 6 Detection Technologies for ecDNA in Breast Cancer

### 6.1 Molecular Biology Approaches

The techniques in molecular biology for detecting ecDNA are undergoing continuous advancement. Presently, sequencing‐based methods, including Circle‐seq, mobilome‐seq, and CIDER‐seq, are extensively utilized. These methodologies facilitate the enrichment and sequencing of ecDNA, thereby enabling a comprehensive characterization of its properties [27]. For example, Circle‐seq analysis of breast cancer cell lines and tissue samples has identified numerous ecDNAs, allowing for an in‐depth examination of their structures and genetic compositions [23]. Furthermore, PCR‐based techniques are employed to detect the presence and amplification of specific genes on ecDNA. For instance, quantitative polymerase chain reaction (qPCR) with gene‐specific primers can evaluate oncogene amplification on ecDNA, contributing to breast cancer diagnosis and prognostic assessment [28]. To improve detection efficiency and accuracy, bioinformatics tools such as ecc_finder, Circle‐Map, and Circle_finder have been developed to accurately identify ecDNA from sequencing data [27]. These tools are instrumental in advancing ecDNA research and its clinical applications in breast cancer.

### 6.2 Imaging Technologies

Imaging technologies present significant potential for the detection of ecDNA. Light microscopy is an essential tool for the identification and characterization of ecDNA, allowing for the visual examination of its morphology and distribution, including structures such as double‐minute chromosomes, through cell staining and microscopic observation [29]. Furthermore, fluorescence‐based imaging techniques, such as fluorescence in situ hybridization (FISH), facilitate the localization and quantitative analysis of specific ecDNAs at the cellular level. This methodology aids in elucidating the spatial distribution and copy number variations of ecDNA within breast cancer cells [30]. Additionally, emerging imaging modalities, such as surface plasmon resonance imaging (SPRi), are being investigated for their potential to detect ecDNA‐associated biomarkers, thereby offering innovative strategies for ecDNA analysis [31].

### 6.3 Potential of ecDNA as a Biomarker in Breast Cancer

ecDNA exhibits considerable potential as a biomarker for breast cancer. Its presence and distinctive characteristics in both tumor tissues and plasma render it valuable for early diagnosis, prognostic evaluation, and treatment monitoring. In patients with breast cancer, the quantity and molecular attributes of plasma ecDNA are closely associated with tumor initiation and progression. The detection of specific genetic alterations in plasma ecDNA, such as gene amplifications, mutations, or methylation patterns, could facilitate noninvasive early diagnosis [25]. Furthermore, ecDNA‐related biomarkers may serve as predictors of patient outcomes. For example, ecDNAs harboring certain oncogenes are linked to poor prognosis, thereby acting as reliable prognostic indicators [2]. In the context of treatment monitoring, dynamic changes in ecDNA levels during therapy can reflect the tumor's response, thereby informing timely adjustments to treatment strategies [30]. Through circular sequencing analysis of 19 cases of breast cancer tissues, researchers discovered ecDNA hotspots in the region of the oncogene ERBB2. The plasma ecDNA levels of these patients were positively correlated with lymph node metastasis (AUC = 0.87), confirming its diagnostic value. This study further indicates that the dynamic changes of ecDNA after chemotherapy can predict cancer recurrence 3 months in advance compared to imaging examinations [23].

In the application of cancer diagnosis, ecDNA differs from other types of DNA. The role of ecDNA in cancer is mainly reflected in its amplification of oncogenes and regulation of gene expression, which makes tumors resistant to treatment, leads to poor prognosis for patients, and accelerates tumor evolution and heterogeneity [15, 19, 32, 33]. In contrast, ctDNA, as a circulating tumor DNA, is a linear DNA fragment released into the bloodstream by apoptotic or necrotic tumor cells. It can be noninvasively detected through liquid biopsy to obtain genomic information of the tumor. It is widely applied in early cancer screening, monitoring of minimal residual lesions, and dynamic tracking of drug‐resistant mutations (such as EGFR T790M mutation) [34, 35, 36]. However, the sensitivity and specificity of ctDNA in the early detection of cancer still face challenges, especially in asymptomatic individuals, where the release of ctDNA may not be sufficient to be detected [37, 38]. mtDNA is mitochondrial genomic DNA in the cytoplasm. Research in cancer mainly focuses on its role in tumorigenesis and metastasis [39]. The fragment characteristics of mtDNA are also regarded as novel biomarkers for multi‐cancer detection, capable of providing significant differences between cancer patients and noncancer controls [40]. It should be noted that mutations or copy number changes in mtDNA are associated with tumor metabolic reprogramming. Although they can be detected in plasma, their tumor specificity is relatively low, and they need to be analyzed in combination with nuclear DNA mutations to distinguish somatic mutations from genetic polymorphisms [41]. Among the three, ecDNA directly promotes the malignant progression of tumors, ctDNA reflects the dynamic burden of tumors, and mtDNA provides metabolic adaptability information. Therefore, in clinical applications, corresponding markers should be selected based on specific scenarios (early diagnosis, prognosis assessment, or mechanism research) [42, 43, 44, 45] (Table 1).

**TABLE 1:** Detection technologies for ecDNA in breast cancer.

Category	Specific technology	Sample type	Advantages	Limitations	Clinical applications
Molecular methods	Circle‐Seq	Tissue/cell lines	Genome‐wide enrichment, structural characterization	Complex workflow, high cost	Oncogenesis studies, ecDNA profiling
	Mobilome‐Seq/CIDER‐Seq	Tissue/plasma	High sensitivity for low‐frequency ecDNA	Requires bioinformatics support	Early diagnosis, recurrence monitoring
	qPCR (gene‐specific)	Tissue/plasma	Rapid, low‐cost, clinical applicability	Limited to known targets	Prognosis assessment (e.g., ERBB2 amplification)
	Bioinformatics tools (ecc_finder, Circle—Map, Circle_finder, etc.)	Sequencing data	Automated identification, high‐throughput	Dependent on sequencing depth/algorithm accuracy	Big‐data mining, molecular subtyping
Imaging techniques	Light microscopy (double minutes)	Cell smears/sections	Visualizes ecDNA morphology	Low resolution, no sequence information	Basic research, rapid screening
	Fluorescence in situ hybridization (FISH)	Cells/tissue	Spatial localization, copy number quantification	Limited to predefined targets	Targeted therapy selection (e.g., MYC amplification)
	Surface plasmon resonance imaging (SPRi)	Plasma/body fluids	Label‐free real‐time detection, high specificity	Expensive equipment, limited clinical validation	Novel biomarker exploration
Biomarker utility	Plasma ecDNA analysis	Liquid biopsy	Noninvasive dynamic monitoring	Low abundance, background noise	Early diagnosis, predicting prognosis, treatment response

## 7 Therapeutic Strategies

### 7.1 Drug Development

The development of pharmacological agents targeting ecDNA has emerged as a highly active area of research in the treatment of breast cancer. In a recent study, researchers used the DNAscent technique to reveal that the replication fork velocity of ecDNA decreased by 6.5% (*p* < 0.01), making it vulnerable to selective clearance by replication stressors such as hydroxyurea (HU), indicating the vulnerability of ecDNA replication stress [7]. Utilizing replication stress to specifically clear ecDNA provides new ideas and strategies for the treatment of diseases. Given the pivotal role of ecDNA in tumorigenesis and resistance to treatment, the creation of therapeutics specifically aimed at ecDNA presents considerable promise. Current research endeavors are concentrated on disrupting the replication and transcription of ecDNA, as well as its interactions with chromosomes, to inhibit tumor progression. For example, studies have indicated that certain topoisomerase inhibitors can interfere with the replication and maintenance of ecDNA, thereby reducing its content and suppressing the proliferation of cancer cells [9]. Since the maintenance of ecDNA requires DDR, especially the alt‐NHEJ pathway, which involves a series of key proteins such as LIG3 and POLθ, they play a crucial role in repairing DSB. Through a series of experiments, researchers have confirmed that inhibiting key factors in the alt‐NHEJ pathway (such as LIG3) can disrupt the cyclization process of ecDNA, leading to a significant decrease in ecDNA levels [9]. Furthermore, efforts are underway to develop drugs that target specific oncogenes harbored by ecDNA, with the aim of mitigating their oncogenic effects by inhibiting oncogene expression. In cases of HER2‐negative breast cancer with ecDNA‐driven CCND1 amplification, CDK4/6 inhibitors showed reduced efficacy (HR = 2.1, risk of progression). This highlights ecDNA as a biomarker of drug resistance and promotes the development of ecDNA‐targeted combination therapies [2]. Nonetheless, several challenges persist in the development of ecDNA‐targeted therapies. Critical issues include enhancing drug specificity and efficacy against ecDNA, as well as overcoming potential drug resistance [46]. Furthermore, studies have shown that the DNA damage repair mediator pRPA2‐S33—a protein capable of binding to single‐stranded DNA—shows transcription‐dependent localization enrichment on ecDNA, accompanied by an increase in DNA double‐strand breaks and activation of the S‐phase checkpoint kinase CHK1. In tumor cells carrying ecDNA, inhibiting CHK1 through genetic means or drugs can trigger extensive and selective tumor cell death. Based on this, Professor Paul Mischel's team developed a CHK1 inhibitor, BBI‐2779, which is highly selective, potent, and has good oral bioavailability. This compound can specifically target and eliminate tumor cells containing ecDNA. In gastric cancer models carrying FGFR2 gene ecDNA amplification, BBI‐2779 not only significantly inhibited tumor growth but also effectively blocked the acquired resistance to the pan‐FGFR inhibitor infigratinib mediated by ecDNA, thereby inducing durable and effective tumor regression in mice. These findings suggest that transcription‐replication conflicts can serve as the molecular basis for ecDNA‐directed therapy, achieving precise cancer treatment by triggering synthetic lethal effects [19]. A systematic analysis was conducted on 19 cases of breast cancer tissues and 17 cases of adjacent normal tissues using whole‐genome Circle‐seq technology. The researchers found that ecDNA was significantly enriched in 7 specific “hot spot genes” (HSGs), and several types of ecDNA carrying miRNA (eccMIR) were identified. Related to the malignant progression of tumors, these functional elements, as dynamic repositories of carcinogens, highlight their dual potential as clinical biomarkers and therapeutic targets [23].

Despite advancements, toxicities and side effects continue to pose significant challenges in ecDNA‐targeted therapy. Inhibition of DNA damage repair pathways, such as CHK1, can selectively eliminate ecDNA‐positive tumor cells. However, this approach may also induce substantial toxicity in normally proliferating cells, such as bone marrow cells, which depend on the CHK1 checkpoint for maintaining genomic stability. This can result in severe hematological adverse reactions and other off‐target effects [19]. Additionally, the dynamic nature of ecDNA and its uneven distribution during cell division can lead to quantitative alterations during treatment, thereby increasing the complexity and unpredictability of therapeutic outcomes [33, 47]. A further major challenge in ecDNA‐targeted therapy is the difficulty patients face in making informed decisions. This is due to the diverse manifestations and mechanisms of action of ecDNA across different cancer types, as well as the presence of complex subclonal dynamic evolution within ecDNA‐positive tumors. Even if clones with high levels of ecDNA are eliminated in the initial stages of treatment, clones with low‐copy or ecDNA‐negative profiles, referred to as “evolutionary reserve” clones, may rapidly proliferate under the selective pressure of treatment, resulting in drug resistance and disease recurrence. Accurately identifying and selecting patients suitable for ecDNA‐targeted therapy has emerged as a significant challenge [48]. Current research indicates that the detection of ecDNA within tumor tissues can optimize patient selection, thereby ensuring the inclusion of individuals most likely to benefit from novel treatments in clinical trials [48]. However, this process necessitates the use of advanced detection technologies and precise molecular typing techniques to ensure the accurate identification and characterization of ecDNA [43, 49]. Future research must further investigate the biological characteristics and therapeutic targets of ecDNA to develop safer and more effective treatment strategies [29, 50].

### 7.2 ecDNA‐Based Personalized Treatment Strategies

The development of personalized treatment regimens based on ecDNA characteristics constitutes a pivotal advancement in precision medicine for breast cancer. By examining ecDNA derived from tumor tissues or plasma in breast cancer patients—including the oncogenes it harbors, its amplification patterns, and associated molecular features—clinicians can select more tailored therapeutic drugs and strategies. For instance, patients with ecDNA harboring specific oncogene amplifications may benefit from targeted therapies directed against those oncogenes. In cases exhibiting high ecDNA loads associated with treatment resistance, the combination of ecDNA‐targeting drugs (to inhibit ecDNA functionality) with conventional chemotherapy may enhance therapeutic efficacy [6]. Moreover, the integration of machine learning to analyze large‐scale ecDNA datasets from patients could refine predictive models, thereby providing robust support for personalized treatment decision‐making [51].

### 7.3 Integration of ecDNA With Breast Cancer Immunotherapy

The integration of ecDNA and immunotherapy constitutes a novel approach in the treatment of breast cancer. The presence of ecDNA has the potential to affect tumor cell immunogenicity and immune evasion mechanisms, indicating that targeting ecDNA could enhance tumor responsiveness to immunotherapy. Suppressing the expression of genes associated with ecDNA may alter the tumor immune microenvironment, thereby facilitating immune cell infiltration and promoting the destruction of tumor cells [20]. Conversely, employing ecDNA as targets for vaccine development could lead to the creation of ecDNA‐based cancer vaccines that stimulate host immune responses, representing an innovative immunotherapeutic strategy. For example, presenting specific antigens derived from ecDNA to the immune system could elicit targeted immune responses against tumor cells [52] (Table 2).

**TABLE 2:** Therapeutic strategies targeting ecDNA in breast cancer.

Strategy	Approach/agent	Mechanism	Research progress	Key challenges	Clinical potential
Targeting ecDNA replication and maintenance (preclinical)	Topoisomerase inhibitors	Suppress ecDNA replication and maintenance by interfering with DNA topology.	Reduces ecDNA content in vitro, inhibits cancer cell proliferation.	Lack of ecDNA specificity, potential systemic toxicity.	Synergistic effects with conventional chemotherapy.
	Inhibitors of alt‐NHEJ pathway (e.g., targeting LIG3/POLθ)	Disrupt the alt‐NHEJ DNA repair pathway, which is crucial for the circularization and maintenance of ecDNA.	Inhibition of key factors (e.g., LIG3) disrupts ecDNA cyclization, leading to a significant decrease in ecDNA levels.	Identifying specific and potent inhibitors; potential compensatory repair mechanisms.	Overcoming ecDNA‐mediated therapy resistance.
	CHK1 inhibitors (e.g., BBI‐2779)	Exploit transcription‐replication conflicts on ecDNA. Inhibit CHK1 to induce synthetic lethality selectively in ecDNA‐positive cells.	BBI‐2779 (a selective CHK1 inhibitor) specifically eliminates ecDNA+ tumor cells, inhibits tumor growth, and blocks ecDNA‐mediated acquired resistance to targeted therapies (e.g., FGFR inhibitors) in preclinical models.	Managing on‐target toxicity; identifying predictive biomarkers for patient selection.	Precision therapy for ecDNA+ cancers; overcoming and preventing acquired resistance.
	Replication stress inducers (e.g., Hydroxyurea—HU)	Exploit the disorganized and vulnerable replication forks on ecDNA.	ecDNA replication fork velocity is reduced (6.5%, *P* < 0.01), making it susceptible to selective clearance by HU.	Achieving tumor‐specific targeting; narrow therapeutic window.	Provides a rationale for using existing replication stress‐inducing agents.
Targeting oncogenes on ecDNA (clinical trial)	Oncogene‐specific targeted agents (e.g., anti‐ERBB2)	Directly inhibit the function or signaling of oncogenes amplified on ecDNA (e.g., ERBB2, MYC).	Effective for specific amplifications (e.g., ERBB2+).	ecDNA evolution and heterogeneity can lead to drug resistance.	Subtype‐specific therapy.
	CDK4/6 inhibitors	Target cell cycle drivers amplified on ecDNA (e.g., CCND1).	Reduced efficacy (HR = 2.1, progression risk) in HER2‐ breast cancer with ecDNA‐driven CCND1 amplification, highlighting ecDNA as a resistance biomarker.	Intrinsic and acquired resistance.	Necessitates combination strategies or patient stratification based on ecDNA status.
Personalized therapy based on ecDNA profiling (preclinical)	ecDNA profiling‐guided treatment	Match therapeutic drugs to the specific oncogenes and amplification patterns carried by ecDNA from patient tumors or liquid biopsies.	Machine learning models are being developed to predict drug response based on ecDNA features.	Tumor heterogeneity; dynamic changes in ecDNA during treatment; technical standardization for clinical use.	Optimized treatment allocation; real‐time adjustment of therapy.
	Combination: ecDNA‐targeting agents + chemotherapy	EcDNA‐targeting agents suppress ecDNA‐mediated resistance mechanisms, sensitizing tumors to conventional chemotherapy.	Preclinical synergy demonstrated.	Defining optimal drug combinations and sequences; managing combined toxicity.	Overcoming therapy resistance (e.g., in TNBC).
Combined immunotherapy (preclinical)	Targeting ecDNA‐mediated immune evasion	Reverse immunosuppression caused by ecDNA, for example, by restoring antigen presentation or modulating the tumor immune microenvironment.	Preclinical evidence suggests ecDNA affects immune gene expression and T cell infiltration.	Complex mechanisms; lack of specific agents targeting these pathways.	Potential to enhance the efficacy of existing immunotherapies (e.g., PD‐1/PD‐L1 inhibitors).
	ecDNA neoantigen vaccines	Utilize unique antigens derived from ecDNA sequences to stimulate a host immune response against the tumor.	Anti‐tumor activity demonstrated in murine models with vaccines targeting oncogenes (e.g., EGF, VEGF).	Identifying immunogenic ecDNA neoantigens; low antigen presentation efficiency.	Novel immunotherapy development.

## 8 Controversies and Challenges

Despite advancements in the study of ecDNA in breast cancer, controversies persist regarding its definitive role. Some research indicates that while ecDNA is linked to tumor initiation and progression, it may not serve as a direct driver of tumorigenesis but rather as an indicator of genomic instability within tumors [15]. Moreover, the mechanisms by which ecDNA operates across various breast cancer subtypes remain inadequately understood, with studies yielding inconsistent results. For instance, certain investigations reveal a strong correlation between ecDNA and tumor invasion or metastasis, whereas others report less significant associations, potentially attributable to sample heterogeneity or methodological discrepancies [24]. Consequently, further research is imperative to elucidate the specific role of ecDNA in the pathogenesis of breast cancer.

The technical sensitivity and standardization of ecDNA detection encounter significant challenges. Currently, ecDNA detection predominantly depends on whole genome sequencing (WGS) and its derivative methodologies, including ATAC‐seq and scCircle‐seq [42, 53, 54]. Nevertheless, short‐read sequencing technologies are inherently limited in their capacity to analyze the intricate circular structures and repetitive sequences characteristic of ecDNA. These limitations often result in an inability to accurately identify connection breakpoints and structural variations [55]. Although advancements in long‐read sequencing technologies, such as Nanopore, and single‐cell multi‐omics approaches, like scGTP, have demonstrated potential in enhancing detection sensitivity, they are still hindered by issues such as high costs, complex operational requirements, nonstandardized bioinformatics analysis processes, and the absence of standardized determination thresholds. These challenges impede the comparability and integration of results across different platforms and laboratories [55]. Additionally, radiology‐based identification methods suffer from low throughput and reliance on subjective judgment, rendering them unsuitable for large‐scale clinical testing. In the interim, substantial discrepancies exist among various studies regarding the sample collection, processing, enrichment, and detection methodologies for ecDNA, thereby complicating the comparison of results [56]. Factors such as the site, timing, and method of sample collection can significantly influence the content and molecular characteristics of ecDNA. This pronounced spatial–temporal diversity can result in false‐negative outcomes in single biopsies or localized sampling, which may not accurately represent the comprehensive tumor profile [57, 58]. Consequently, this limitation significantly constrains the clinical utility of ecDNA as a reliable biomarker. Even with advancements in liquid biopsy technology, the detection of ecDNA in plasma is influenced by several factors, including its release kinetics, degree of degradation, and the proportion of dominant clones. Currently, it remains uncertain whether the sensitivity of ecDNA detection surpasses that of traditional ctDNA. Furthermore, the absence of standardized quality control measures and reference materials poses significant challenges to the standardization of ecDNA testing. Therefore, establishing a standardized and fully controllable ecDNA detection and quality system is of great significance for accurately assessing its role in breast cancer and promoting clinical transformation.

Additionally, ecDNA research presents significant ethical considerations. It is imperative that sample collection and usage adhere to protocols that ensure patient informed consent while safeguarding privacy and rights. Given that ecDNA testing may disclose genetic information, there is a risk of misuse leading to discrimination in insurance or employment [59]. Clinical trials and therapeutic interventions involving ecDNA must adhere strictly to ethical guidelines, with a thorough assessment of risks and benefits to ensure patient safety. Furthermore, as international collaboration in ecDNA research expands, harmonizing ethical standards across various regions and countries is essential to foster responsible and sustainable advancements in this field.

## 9 Future Perspectives of ecDNA Research in Breast Cancer

See Figure 2.

**FIGURE 2:**
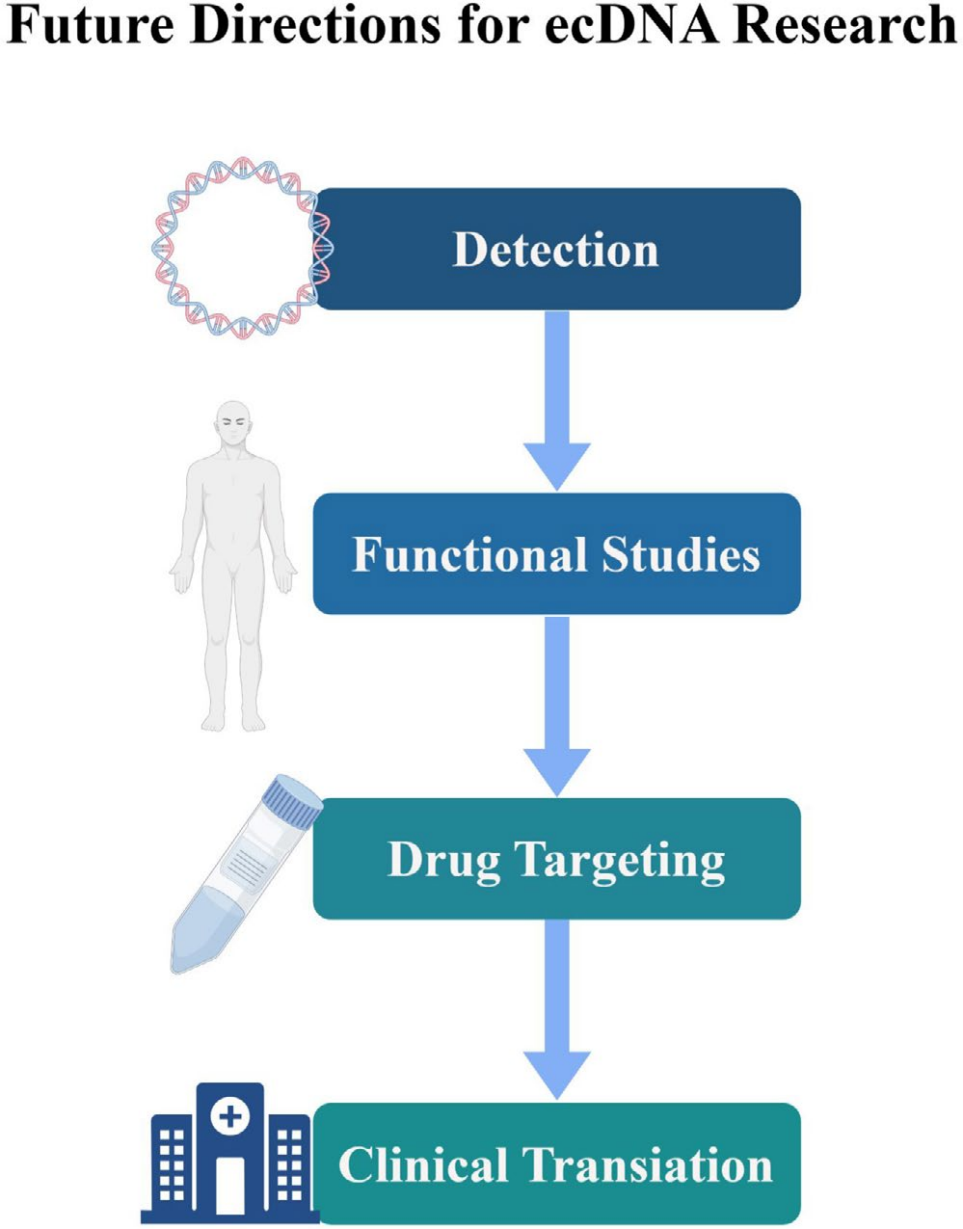
Future directions for advancing ecDNA research in breast cancer. Key focus areas include: Detection, emphasizing the need for developing more sensitive and standardized methods to identify and characterize ecDNA in both tissue and liquid biopsies; Functional Studies, aimed at elucidating the precise molecular mechanisms of ecDNA biogenesis, maintenance, and its role in tumor evolution and immune modulation; Drug Targeting, focused on designing novel therapeutic strategies that exploit ecDNA‐specific vulnerabilities, such as its distinct replication and repair pathways; and Clinical Translation, which involves validating ecDNA as a biomarker for diagnosis, prognosis, and therapy response monitoring, and integrating these findings into personalized treatment approaches to improve patient outcomes.

### 9.1 Technological Advancements

Future research on ecDNA is anticipated to yield significant technological advancements. Detection technologies are expected to undergo continuous optimization and innovation, enhancing the sensitivity, specificity, and throughput of ecDNA analysis. For example, advancements in next‐generation sequencing are likely to enable a more comprehensive characterization of ecDNA, encompassing its structure, methylation patterns, and interactions with other molecules [60]. Concurrently, imaging technologies are projected to be refined to permit real‐time, high‐resolution monitoring of ecDNA dynamics within cells, thereby offering deeper insights into its biological functions and mechanisms [29]. Furthermore, gene editing tools, such as the CRISPR‐Cas system, will provide robust platforms for functional studies of ecDNA. Precise editing of ecDNA will facilitate detailed investigations into its specific roles in the initiation and progression of breast cancer. Researchers can use CRISPR‐Cas9 technology to construct ecDNA research models. By precisely cutting out specific DNA fragments on chromosomes (including segments of oncogenes) and utilizing the cell's own repair mechanism, they can form ecDNA by connecting them “head to tail” and cyclizing them. This method can construct stable cell lines carrying specific ecDNA in the laboratory, providing a strictly matched control model for studying their replication, maintenance, and function. Researchers can also directly target and edit ecDNA. Theoretically, designing guide RNA (gRNA) targeting the specific ligating sequences of ecDNA or specific oncogenes (such as MYC, EGFR) and forming complexes with Cas enzymes can specifically cleave ecDNA, induce its degradation, or render it unable to replicate. Because ecDNA lacks chromosomal protective structures such as centromeres, this targeted cleavage may lead to its effective clearance in cells undergoing mitosis. In addition, catalytically inactivated Cas9 (dCas9) can fuse with epigenetic effectors and others, target ecDNA, and introduce specific epigenetic modifications such as adding inhibitory histone markers H3K9me3 or H3K27me3, thereby efficiently silencing the expression of oncogenes on it without altering its DNA sequence [9]. In addition, CRISPR‐based gene screening (CRISPR screen) can be used to systematically screen key genes related to the maintenance, replication, and function of ecDNA. New therapeutic targets can be discovered by screening for gene knockout that causes synthetic lethality or ecDNA loss in ECDNA‐positive cells across the entire genome. CRISPR technology can also be used for the detection of ecDNA. There is a patented technology that utilizes the CRISPR‐Cas9 system to specifically identify and cut the target sequence on ecDNA and combines rolling loop amplification (RCA) and the CRISPR‐Cas14 system for signal amplification, achieving highly sensitive detection of ecDNA at concentrations as low as 1 fM. Moreover, there is no need to pre‐digest linear genomic DNA, which shortens the detection time [61].

### 9.2 Potential Applications

Research on ecDNA presents extensive potential applications in the management of breast cancer. In addition to their role as biomarkers for early diagnosis, prognostic assessment, and treatment monitoring, ecDNA may unveil novel therapeutic targets and strategies. Advances in the development of ecDNA‐targeted drugs are expected to yield more effective treatment options for breast cancer patients [62]. Moreover, the integration of ecDNA‐based approaches with immunotherapy, gene therapy, and other treatment modalities demonstrates considerable promise. The synergistic effects of combining multiple therapeutic strategies could substantially enhance treatment outcomes and improve patient prognosis. Furthermore, ecDNA research is poised to deepen our understanding of breast cancer pathogenesis, thereby informing the development of innovative prevention strategies.

### 9.3 Development Trends

Artificial intelligence (AI) and machine learning (ML) have demonstrated considerable promise in the realm of biomarker discovery, particularly in the context of analyzing large‐scale sequencing data [51, 63]. Within the field of cancer research, AI and ML have been extensively utilized to identify and examine ecDNA. For example, machine learning techniques can discern amplification patterns from large‐scale whole exome sequencing data and associate these patterns with clinical and molecular characteristics. This approach not only uncovers the mutually exclusive relationship between ecDNA amplification and microsatellite instability but also provides clinical evidence supporting the efficacy of immunotherapy [51]. Furthermore, the integration of machine learning in miRNA cancer research has underscored its pivotal role in biomarker discovery and therapeutic targeting. By employing deep learning technologies, researchers can efficiently identify key miRNAs across various cancers and construct prognostic models [64]. Despite the significant advantages offered by AI and ML, their application is still confronted with challenges such as complex datasets, high levels of noise, and susceptibility to overfitting [65]. The emergence of explanatory AI has, to some extent, improved the interpretability of models, facilitating the identification of meaningful discoveries prior to validation [65]. Furthermore, machine learning excels in the integration of multi‐omics data, systematically elucidating disease mechanisms and advancing precise diagnosis and personalized treatment by combining genomic, transcriptomic, proteomic, and metabolomic data [66, 67]. Collectively, AI and machine learning are pivotal in the identification of biomarkers, such as ecDNA. Despite challenges related to data complexity and interpretability, the potential applications of these technologies in biomedicine remain extensive, supported by advancements in algorithm optimization and multi‐dimensional data integration [68, 69].

Furthermore, in the evolving landscape of ecDNA research, two critical areas demand urgent advancement: the standardization of detection technologies and the clinical translation of dynamic monitoring methods. Establishing a standardized ecDNA detection framework is essential for its integration into clinical practice. Currently, ecDNA detection via tissue biopsy is constrained by tumor heterogeneity and the invasive nature of the procedure, complicating the ability to perform repeated sampling necessary for monitoring tumor evolution dynamically. Although liquid biopsy presents a noninvasive alternative with promising potential, its clinical implementation encounters significant challenges. Detecting ecDNA in plasma necessitates exceptionally high sensitivity and specificity [70], particularly in distinguishing linear ctDNA from apoptotic cells and intact circular ecDNA molecules [71]. This necessitates the development of enrichment techniques that target the cyclic characteristics of ecDNA, such as capture methods based on rolling loop amplification or topological properties, alongside the optimization of bioinformatics processes to accurately identify its cyclic structure and chimeric junctions. Additionally, the comparison of results across various detection platforms, such as droplet digital PCR (ddPCR) and next‐generation sequencing (NGS), as well as between different laboratories, necessitates the urgent development of standardized reference materials. Examples of such materials include synthetic circular DNA standards or standard cell lines with known extrachromosomal DNA (ecDNA) sequences and structures, which are essential for quality control and consistent validation across laboratories [72]. Advancing these standardization initiatives will substantially improve the reproducibility and comparability of ecDNA testing, thereby establishing a foundation for future large‐scale clinical research.

Secondly, the dynamic monitoring of disease progression and the evaluation of treatment response through ecDNA represent a promising yet insufficiently explored area of research. Owing to its nonchromosomal genetic properties, ecDNA undergoes highly dynamic alterations during tumor evolution, rendering it an ideal biomarker for tracking clonal evolution and the development of drug resistance. Nevertheless, there remains a paucity of systematic investigations into methodologies for high‐frequency and high‐sensitivity tracking of dynamic changes in ecDNA content, structural variations, and associated genes throughout the treatment process. Future research should focus on developing strategies for longitudinal sample collection, such as continuous liquid biopsy, and integrating deep sequencing with single‐molecule techniques to elucidate the relationship between ecDNA evolution and clonal selection under therapeutic pressure. For example, it is imperative to investigate whether fluctuations in ecDNA copy number and structural rearrangements can serve as predictors of secondary drug resistance in targeted therapy or chemotherapy, and whether ecDNA can be utilized as an early indicator for assessing response to immunotherapy [73, 74]. Addressing these scientific challenges necessitates not only technological innovation but also the backing of prospective clinical cohorts to validate their dynamic clinical relevance, ultimately facilitating the optimization of individualized treatment strategies.

In the process of translating research findings on ecDNA into clinical practice, it is imperative to establish a comprehensive translational framework [75]. Initially, during the discovery phase, multi‐omics technologies are employed to systematically identify ecDNA across various cancer types, while artificial intelligence is utilized to investigate its association with tumorigenesis, metastasis, or drug resistance. The subsequent phase involves validation, wherein standardized detection methodologies, such as sequencing‐based or optical imaging techniques, are applied to retrospective large cohorts to substantiate the clinical relevance of potential ecDNA biomarkers. Finally, during the biomarker development phase and the standardization of detection methods, the validated ecDNA characteristics are converted into clinically applicable assays, such as plasma liquid biopsy or digital PCR detection, and their analytical performance is rigorously validated. Ensure the sensitivity, specificity, and repeatability of the biomarkers. In the subsequent clinical trial integration phase, the utility of ecDNA biomarkers is assessed through prospective clinical trials. These trials aim to evaluate their efficacy in predicting responses to specific targeted therapies or immunotherapies, as well as in dynamically monitoring the development of treatment resistance. Ultimately, patient stratification and precision treatment are implemented. The overarching objective is to categorize patients based on their ecDNA status to inform clinical decision‐making. For example, patients exhibiting positive ecDNA may be directed towards more aggressive treatment regimens or considered for inclusion in clinical trials exploring novel therapies that target the maintenance mechanisms of ecDNA.

At present, research on ecDNA is increasingly characterized by a trend towards enhanced international collaboration and multidisciplinary integration. Given that ecDNA studies encompass a range of disciplines, including oncology, genetics, molecular biology, and imaging science, global cooperation is essential for the integration of resources and the fostering of interdisciplinary innovation. Through international partnerships and multidisciplinary efforts, research teams across the globe can share data, technologies, and expertise to collectively address the challenges inherent in ecDNA research. For instance, large‐scale international multicenter clinical trials may be conducted to validate the efficacy and safety of ecDNA‐related biomarkers and therapeutic strategies. In the future, ecDNA research is expected to enhance international collaboration and data sharing, expedite its clinical translation through AI‐driven insights, and ultimately enable precise diagnosis, treatment selection, and patient management based on ecDNA, thereby delivering tangible clinical benefits to patients with malignant tumors, including breast cancer.

## 10 Conclusions

Current research indicates that ecDNA plays a crucial role in the initiation, progression, diagnosis, and treatment of breast cancer. As a distinct form of DNA, ecDNA can harbor oncogenes, thereby enhancing gene expression and facilitating tumor progression. The mechanisms underlying ecDNA formation in breast cancer are complex, involving multiple factors such as aberrant DNA damage repair and chromosomal rearrangements. Presently, the presence of ecDNA is strongly associated with the prognosis, invasiveness, and treatment resistance of breast cancer, with its distribution and function differing across various breast cancer subtypes. Numerous molecular and imaging techniques have been employed for the detection of ecDNA, and its detectability in plasma suggests its potential utility as a biomarker for liquid biopsy. Nonetheless, the precise molecular mechanisms underlying ecDNA formation, its dynamic alterations across different breast cancer subtypes, and the specific pathways involved in its immunomodulatory functions remain inadequately understood. Furthermore, the standardization of detection methods and the resolution of ethical concerns present ongoing challenges in this area of research. Current research predominantly addresses the biological characteristics, detection methodologies, and targeted therapeutic strategies associated with ecDNA. Nonetheless, ecDNA is anticipated to emerge as a significant biomarker for early diagnosis, prognosis evaluation, and dynamic monitoring of breast cancer. The advancement of targeted therapies aimed at ecDNA replication mechanisms, transcription‐replication conflicts, or associated DNA repair pathways, alongside their integration with immunotherapy and novel detection technologies, offers a promising avenue for overcoming drug resistance and achieving personalized treatment for breast cancer.

## Author Contributions

**Xiaodan Zhu:** conceptualization (equal), methodology (lead), supervision (equal), funding acquisition (lead), visualization (equal), writing – original draft (equal). **Yiyang Qian:** conceptualization (equal), software (lead), data curation (lead), visualization (equal), writing – original draft (equal). **Quan Tang:** software (supporting), writing – review and editing (equal). **Jiahui Li:** software (supporting), writing – review and editing (equal). **Yuqing Geng:** data curation (supporting), writing – review and editing (equal). **Shixia Gong:** data curation (supporting), supervision (equal), writing – review and editing (equal). **Chunhui Jin:** supervision (equal), writing – review and editing (equal).

## Ethics Statement

The authors have nothing to report.

## Consent

The authors have nothing to report.

## Conflicts of Interest

The authors declare no conflicts of interest.

## Data Availability

Data sharing not applicable to this article as no datasets were generated or analyzed during the current study.
