# Correction to “Research Advances of Extrachromosomal Circular DNA in Breast Cancer”

**DOI:** 10.1002/cam4.72433

**Published:** 2026-11-02

X. Zhu , Y. Qian , Q. Tang , et al., “Research Advances of Extrachromosomal Circular DNA in Breast Cancer,” Cancer Medicine 14, no. 19 (2025): e71285, 10.1002/cam4.71285.PMC1249827741051137

Reference 21, 22 were included incorrectly, and have been updated to the following reference:

Bailey C, Pich O, Thol K, et al. “Origins and Impact of Extrachromosomal DNA,” *Nature* 635, no. 8037 (2024): 193–200.

Reference 55 was included incorrectly, and has been updated to:

Chang L, Deng E, Wang J, Zhou W, Ao J, Liu R, Su D, Fan X. “Single Cell Third Generation Sequencing Based Multi Omics Uncovers Gene Expression Changes Governed by ecDNA and Structural Variants in Cancer Cells,” *Clinical and Translational Medicine* 13, no. 8 (2023): e1351.

We apologize for these errors.

